# Assessment of Blood Pressure Variability in Postmenopausal Women

**DOI:** 10.7759/cureus.29471

**Published:** 2022-09-22

**Authors:** Ayushi Jain, Sunil Kumar, Sourya Acharya, Ruchita Kabra, Rucha Sawant

**Affiliations:** 1 Department of Medicine, Jawaharlal Nehru Medical College, Datta Meghe Institute of Medical Sciences (Deemed to be University), Wardha, IND; 2 Internal Medicine, Jawaharlal Nehru Medical College, Datta Meghe Institute of Medical Sciences (Deemed to be University), Wardha, IND

**Keywords:** cardiovascular, aging, menopause, variation, blood pressure

## Abstract

Introduction: Increase in blood pressure is the leading cause of comorbidity, especially cardiovascular and cerebrovascular disease, but its variability represents an independent risk factor for the same in menopausal women. Dramatic decrease of estrogen production in menopause interferes with many metabolic pathways leading to insulin resistance, increased body mass index (BMI) and dyslipidemia hence cardiovascular morbidity and mortality. In this study an assessment of the variability of blood pressure in postmenopausal women and its association with BMI have been highlighted.

Method: In this cross‐sectional study, a total of 175 postmenopausal females more than 50 years of age who were admitted to or visited medicine wards with no known major medical condition were enrolled between June and July 2019. Recording of both diastolic and systolic blood pressure of postmenopausal women was done at 6 am, 12 pm and 7 pm manually by mercury sphygmomanometer. In addition, BMI, neck circumference, and lipid profile values were also recorded.

Results: Variation of both systolic and diastolic blood pressure at 6 am, 12 pm and 7 pm in association with various parameters like BMI (more than 25 kg/m^2 ^) were 128.57 ± 12.68/78.93 ± 9.56; 124.64 ± 12.32/77.14 ± 5.35 and 124.29 ± 12.60/75.36 ± 6.37 mmHg respectively. Neck circumference (>32 cm) was similar at 128.89 ± 13.49/79.86 ± 8.47; 124.58 ± 12.99/77.08 ± 7.59 and 125.56 ± 12.21/76.94 ± 7.62 mmHg respectively.

Conclusion: BMI and neck circumference as well as triglyceride and cholesterol increase may all independently contribute to the blood pressure variability in menopausal women.

## Introduction

Palpitations, hot flashes, family history of heart disease, shortness of breath, chest heaviness, headache, lightheadedness or dizziness, diabetes or dyslipidemia, and obstructive sleep apnea are important symptoms and conditions which should not be taken lightly as they may be an indication of high blood pressure (BP) in menopausal women. 

Blood pressure variability (BPV) represents an independent risk factor for cardiovascular diseases especially in postmenopausal women where the hormonal protective effect is decreased [1]. Though the basic pathophysiology of BPV is not fully clear, alterations in sympathetic regulative functions have been postulated. This may predispose individuals to abnormally high blood pressure fluctuations [2,3]. This may be due to baroreflex dysfunction, as a result of atherosclerotic arterial wall stiffening, which hinders the cardiovascular system to regulate stress-induced blood pressure oscillations [3].

Postmenopausal women have an increased risk of cardiovascular and cerebrovascular morbidity and mortality, probably due to the appearance of major risk factors such as dyslipidemia, diabetes, obesity and hypertension [4,5]. Many metabolic pathways may be affected in postmenopausal women due to a decrease in estrogen production which may lead to insulin resistance, increased body mass index (BMI) and dyslipidemia [6-10]. Whether menopausal changes in sympathetic haemodynamic regulation lead to enhanced blood pressure variability is not clear. In addition, variability of BP, especially a rise during night hours, is correlated with more target organ complications. Postmenopausal females are more affected compared with males and premenopausal females in nocturnal nonreduction of BP [11-14].

There are not enough data on the assessment of BP variability in postmenopausal women in India. The research question for the current study was to assess the variability of blood pressure in postmenopausal women and to compare this variability of blood pressure with special reference to BMI, dyslipidemia and neck circumference.

## Materials and methods

Study setting

This cross-sectional study was carried out in the Department of Medicine, Acharya Vinoba Bhave Rural Hospital and Jawaharlal Nehru Medical College attached with Datta Meghe Institute of Medical Sciences, Wardha.

Study population

All consecutive postmenopausal females of more than 50 years of age admitted/visited in medicine wards with no known major medical condition.

The sample size was calculated by the formula n = Z (⌊1−α /2⌋) ². p (1-p)d² where, Z (⌊1−α /2⌋ is the level of significance at 5% i.e. 95% confidence interval = 1.96; P = prevalence of hypertension in postmenopausal women = 33% = 0.33 [7] and d = derived error of margin = 7% = 0.07; therefore, n = (1.96)². 0.33. 0.67 = 173.26 ≈ 175 (0.07)². Therefore, the number of patients needed for the study is 175.

Selection criteria

All consecutive postmenopausal females admitted/visited in medicine wards of more than 50 years of age with no known major medical conditions like diabetes mellitus, chronic kidney disease, thyroid disorders, or ischemic heart disease were included in the study. Written informed consent was taken from all participants for subsequent study procedures. The study was approved by the Institutional Ethics Committee (Reference no. DMIMS(DU)/IEC/2019/8010).

Data collection method

For a period of two months beginning June 2019, all prospectively enrolled postmenopausal female patients were studied.

Apart from noting demographic features like age, education, social status, BMI and the recording of both diastolic and systolic blood pressure of postmenopausal women were done at 6 am, 12 pm and 7 pm. All anthropometric measurements (weight, height, and neck circumference) were performed according to WHO guidelines [15,16]. Weight was measured in to the nearest 100 grams using calibrated spring balance without heavy clothing and barefooted with the same standardized weighing machine for all patients. Height was measured to the nearest centimeter, barefooted using a stadiometer. Neck circumference was measured to the nearest 0.1 centimeter just below the laryngeal prominence (Adam’s apple) in all the participants [17]. All circumferences were taken with the subjects standing upright, with shoulders relaxed using flexible measuring tapes. Blood pressure was recorded in the sitting position after five minutes of rest using standard mercury manometer. Hypertension was diagnosed when systolic BP (SBP) > 140 mmHg and/or diastolic BP (DBP) > 90 mmHg or a known hypertensive patient [4]. BMI (weight in Kg)/(height in meters)^2^ was calculated and overweight and obesity was considered when BMI was between 25-29.9 kg/m^2^ and more than 30 kg/m^2^ respectively.

Statistical analysis

We performed the Wilcoxon Test and Spearman Correlation in order to assess possible correlations between mean BP and BP variability data with BMI, neck circumference and lipid profile. Data were expressed as mean value ± SD. P value less than or equal to 0.05 was statistically significant.

## Results

Out of 175 patients, 122 (69.7%) were illiterate and 127 (72.6%) were from a rural background. The mean BMI was 21.37±4.27 kg/m2 and the mean neck circumference was 31.23±2.28cm. All other parameters are shown in Table 1.

**Table 1:** Baseline characteristics SD- Standard Deviation BMI- Body Mass Index (Diabetes Mellitus included in the table was diagnosed at the time of screening)

Parameters (n=175)	Mean ± SD/N (%)
Age (Years)	60.89 ± 7.22
Education	
Literate	53 (30.3%)
Illiterate	122 (69.7%)
Social Status	
Urban	48 (27.4%)
Rural	127 (72.6%)
BMI (Kg/m2)	21.37 ± 4.27
<25 Kg/m2)	147 (84.0%)
>25 Kg/m2	28 (16.0%)
Neck Circumference (cm)	31.23 ± 2.28
<32 cm	103 (58.9%)
>32 cm	72 (41.1%)
Hypertension (Present)	55 (31.4%)
Diabetes Mellitus (Present)	33 (18.9%)
Hemoglobin (gm/dl)	11.70 ± 1.69
Random Blood Sugar (mg/dl)	113.41 ± 55.34
Total Cholesterol (mg/dl)	181.84 ± 43.06
Serum Triglycerides (mg/dl)	152.41 ± 87.10

Table 2 shows variation of both systolic and diastolic blood pressure at 6 am, 12 pm and 7 pm in association with various parameters like BMI and neck circumference.

**Table 2:** Association between BP in mmHg at 6 am, 12 pm and 7 pm Parameters 1: Wilcoxon Test, 2: Spearman Correlation BP-Blood Pressure

Parameter	Systolic BP (mmHg)	p value	Diastolic BP (mmHg)	p value
BMI	6AM		6AM	
		0.044^1^		0.852^1^
<25 Kg/m2)	125.99 ± 15.29		77.82 ± 8.72	
>25 Kg/m2	128.57 ± 12.68		78.93 ± 9.56	
Neck Circumference		0.013^1^		0.023^1^
<32 cm	124.66 ± 15.64		76.70 ± 8.90	
>32 cm	128.89 ± 13.49		79.86 ± 8.47	
	12 PM		12 PM	
BMI		0.464^1^		0.293^1^
<25 Kg/m2)	122.24 ± 13.84		75.78 ± 8.91	
>25 Kg/m2	124.64 ± 12.32		77.14 ± 5.35	
Neck Circumference		0.199^1^		0.217^1^
<32 cm	121.26 ± 13.91		75.24 ± 8.95	
>32 cm	124.58 ± 12.99		77.08 ± 7.59	
	7PM		7PM	
BMI		0.635^1^		0.550^1^
<25 Kg/m2)	126.12 ± 16.11		77.20 ± 11.99	
>25 Kg/m2	124.29 ± 12.60		75.36 ± 6.37	
Neck Circumference		0.986^1^		0.487^1^
<32 cm	126.02 ± 17.62		76.88 ± 13.29	
>32 cm	125.56 ± 12.21		76.94 ± 7.62	

Table 3 shows variation of both systolic and diastolic blood pressure at 6 am, 12 pm and 7 pm in association with serum triglyceride and total cholesterol.

**Table 3:** Correlation between lipid profile and blood pressure variability 1: Wilcoxon Test, 2: Spearman Correlation BP- Blood Pressure

Parameter	Systolic BP (mmHg)	p value	Diastolic BP (mmHg)	p value
Serum Triglycerides (mg/dl) (12PM)	rho = 0.02	0.757^2^	rho = 0	0.953^2^
Total Cholesterol (mg/dl) (12PM)	rho = 0.1	0.175^2^	rho = 0.09	0.237^2^
Total Cholesterol (mg/dl) (7PM)	rho = 0.2	0.009^2^	rho = 0.24	0.002^2^
Serum Triglycerides (mg/dl) (7PM)	rho = 0.07	0.392^2^	rho = 0.07	0.369^2^
Total Cholesterol (mg/dl) (6 AM)	rho = 0.14	0.062^2^	rho = 0.12	0.105^2^
Serum Triglycerides (mg/dl) (6 AM)	rho = 0.03	0.710^2^	rho = 0.09	0.259^2^

## Discussion

The decline in hormones following menopause, especially low estrogen levels, affects blood flow, putting more strain on the heart to ensure proper blood circulation, leading to increased blood pressure [3,4]. Sometimes declining metabolism in menopause makes women less active causing increase in weight gain, which leads to high blood pressure contributing to many morbidities especially cardiovascular disease. Menopausal females are also sensitive to salt and non-restriction of this can lead to water retention due to increased sodium in the body, putting pressure on the blood vessels hence an increase in blood pressure [16].

Increase and variability in blood pressure have more risk of cardiovascular as well as cerebrovascular diseases in menopausal women. However, risk increases with age or the body mass index is not clear. In a study by Zanchetti et al., it was concluded that blood pressure of menopausal women is significantly, though slightly, higher than that in age- and BMI-matched premenopausal women. The author also reported that age and BMI do not affect the incidence of hypertension [11].

Our study showed that BMI and neck circumference were associated positively with SBP and DBP variability at 6 am, 12 pm and 7 pm in postmenopausal women. At 6 am and 12 pm, patients with high BMI and neck circumference had higher blood pressure, but there was dipping of blood pressure at 7 pm. Total cholesterol and triglyceride had also shown some variability in the morning, noon and evening, but they were statistically insignificant. Proposed hypothesis, for haemodynamic changes like blood pressure variation though not clear, it maybe the expression of baroreflex impairment due to menopausal atherosclerosis. Another possibility may be the cardiovascular response to centrally induced sympathetic hyperactivity [12,13].

There are no other studies that have compared the BPV in morning, noon and evening hours. Few studies have shown a significant increase in blood pressure variability in menopausal women when compared to fertile women, even after exclusion of confounding factors, such as aging and BMI [14-17]. Menopausal status and BMI increase may all, independently, contribute to enhanced blood pressure. Few studies have shown importance of neck circumference in predicting obesity and overweight [17]. No other study has compared BPV with neck circumference in postmenopausal women.

One study, in which 24-hour blood pressure of menopausal women was monitored, revealed that diastolic blood pressure was increased during the night hours in the symptomatic group compared to the asymptomatic group. Baroreflex dysfunction was the possible explanation as this causes a peripheral vasoconstriction leading to increases in heartbeat and heart output thence high systolic blood pressure during the day hours. They also showed no variation in systolic blood pressure during the night while sleeping [18].

Limitation

Blood pressure variability was assessed manually, ideally it should be by continuous Holter monitor, being the main limitation of the study. As it was a short-term studentship research training programme, the sample size was small.

## Conclusions

BPV in postmenopausal women is more in the morning and noon but not in the evening. BPV, especially a rise during sleep, may lead to cardiovascular complications like carotid intima thickening and carotid plaque formation. BPV in menopause may be an expression of baroreflex impairment and centrally induced sympathetic hyperactivity. Cholesterol and triglycerides are also correlated with this variability, but not statistically. More studies are required to confirm this hypothesis of the influence of menopausal status on BPV.
